# Mitochondrial respiratory activity and DNA damage in peripheral blood mononuclear cells in borderline personality disorder

**DOI:** 10.1017/S0033291725102493

**Published:** 2025-11-28

**Authors:** Alexander Behnke, Manuela Rappel, Laura Ramo-Fernández, R. Nehir Mavioğlu, Benjamin Weber, Felix Neuner, Ellen Bisle, Matthias Mack, Peter Radermacher, Stephanie H. Witt, Christian Schmahl, Alexander Karabatsiakis, Iris-Tatjana Kolassa

**Affiliations:** 1 Clinical & Biological Psychology, Institute of Psychology and Education, Ulm University, Ulm, Germany; 2 German Center for Mental Health (DZPG), Partner Site Mannheim-Heidelberg-Ulm, Ulm, Germany; 3 German Center for Child and Adolescent Health (DZKJ), Partner Site Ulm, Ulm, Germany; 4 Institute for Anesthesiologic Pathophysiology and Process Engineering, Ulm University Hospital, Ulm, Germany; 5 Department Genetic Epidemiology in Psychiatry, Central Institute of Mental Health, Medical Faculty Mannheim, Heidelberg University, Mannheim, Germany; 6 Department of Psychosomatic Medicine and Psychotherapy, Central Institute of Mental Health, Medical Faculty Mannheim, Heidelberg University, Mannheim, Germany

**Keywords:** borderline personality disorder, biomarker, DNA damage, mitochondrial bioenergetics, oxidative stress

## Abstract

**Background:**

Alterations in the central and peripheral energy metabolism are increasingly recognized as key pathophysiological processes in psychiatric disorders. We investigated mitochondrial respiration and density linked to cellular energy metabolism and oxidative DNA damage in borderline personality disorder (BPD).

**Methods:**

This cross-sectional case–control study compared three groups matched for age and body mass index: women with acute BPD, remitted BPD, and female healthy controls (*n* = 32, 15, 29). Peripheral blood mononuclear cells were investigated for differences in mitochondrial respiration, density, and markers of oxidative DNA damage.

**Results:**

Participants with acute BPD showed significantly reduced and less efficient mitochondrial ATP production compared to both remitted individuals and controls. Mitochondrial coupling and respiration were inversely associated with oxidative DNA damage, although DNA damage levels did not differ significantly across diagnostic groups. Sensitivity analyses indicated that comorbid major depressive episodes and antidepressant use did not account for the results.

**Conclusions:**

These findings indicate mitochondrial alterations accompany acute symptom severity in BPD and may improve with remission. Unraveling causes and consequences of mitochondrial downregulation and its interplay with DNA maintenance in the context of stress and psychopathology could contribute to novel models and treatment strategies in BPD and related severe psychiatric disorders.

## Introduction

Various psychiatric disorders exhibit alterations in energy metabolism, with mitochondrial function and redox biology as key cellular interfaces (Andreazza et al., 2025; Bernard et al., 2025; Henkel et al., 2022; Kim et al., 2019; Mansur, Lee, McIntyre, & Brietzke, 2020; Ni, Ma, & Chung, 2024; Papageorgiou & Filiou, 2024; Sarnyai & Ben-Shachar, 2024). Mitochondria regulate energy production via oxidative phosphorylation (OxPhos) and the tricarboxylic acid (TCA) cycle, contribute to redox signaling and reactive oxygen species (ROS) generation, and influence inflammatory responses (Monzel, Enríquez, & Picard, 2023; Picard & Shirihai, 2022; Picard, Trumpff, & Burelle, 2019). A growing body of evidence highlights changes in mitochondrial dynamics and function in disorders such as bipolar disorder, schizophrenia, and major depressive disorder (MDD) (Andreazza et al., 2025; Bernard et al., 2025; Bisle et al., 2025; Giménez-Palomo, Andreu, et al., 2024; Gumpp et al., 2021; Henkel et al., 2022; Karabatsiakis & Schönfeldt-Lecuona, 2020; Kim et al., 2019; Mansur et al., 2020; Ni et al., 2024; Papageorgiou & Filiou, 2024; Sarnyai & Ben-Shachar, 2024; Zou et al., 2025). In MDD, for instance, decreased OxPhos activity and adenosine triphosphate (ATP) production have been found in various peripheral tissues, including blood immune cells and platelets, alongside alterations in mitochondrial content per cell (Boeck et al., 2018; Gamradt et al., 2021; Gardner & Boles, 2011; Gumpp et al., 2021; Hroudová, Fišar, Kitzlerová, Zvěřová, & Raboch, 2013; Karabatsiakis et al., 2014, 2020; Kuffner et al., 2020; Triebelhorn et al., 2022; Zvěřová et al., 2019). Mitochondrial function also appears sensitive to psychosocial risk factors such as early adversity (Boeck et al., 2016, 2018; Gumpp et al., 2023, 2020; Mavioğlu et al., 2025; Trumpff et al., 2024).

Despite such evidence in other psychiatric conditions, the role of mitochondrial bioenergetics in *Borderline Personality Disorder* (BPD) remains largely unexplored. BPD is a complex psychiatric condition characterized by emotional dysregulation, inconsistent identity, interpersonal difficulties, chronic feelings of emptiness, and heightened risk for self-injury and suicide (Bohus et al., 2021). Its frequent comorbidity and shared symptoms with disorders such as MDD and bipolar disorder suggest overlapping biological mechanisms (Bohus et al., 2021). However, studies on cellular energy metabolism in BPD are scarce (Saccaro, Schilliger, Dayer, Perroud, & Piguet, 2021). Preliminary findings have pointed to reduced cerebral glucose metabolism and altered neurovascular regulation in BPD (Cattarinussi et al., 2022; De La Fuente et al., 1997), potentially reflecting compromised cellular energy metabolism. In the periphery, increased inflammatory signaling and reduced antioxidant defense have been observed (Díaz-Marsá et al., 2012; Kahl et al., 2006, 2005; Lee, Gozal, Coccaro, & Fanning, 2020; MacDowell et al., 2020; Ruiz-Guerrero et al., 2023), yet mitochondrial function has not been directly assessed in this population.

Alongside energy production, mitochondria are key regulators of oxidative stress through their generation of ROS (Cheng et al., 2017; Demine, Renard, & Arnould, 2019; Zhao, Jiang, Zhang, & Yu, 2019). Excessive ROS can overwhelm antioxidant defenses, leading to oxidative damage of proteins, membranes, and nuclear and mitochondrial DNA (Fang et al., 2016; Maynard et al., 2013). Accumulated DNA damage has been implicated in accelerated cellular aging, persistent inflammation, and neuropsychiatric vulnerability (Czarny, Bialek, Ziolkowska, Strycharz, & Sliwinski, 2019; Czarny, Wigner, Gałecki, & Śliwiński, 2018; Fang et al., 2016; Giménez-Palomo, Andreu, et al., 2024; Kidane et al., 2014; Kim et al., 2019; Maynard et al., 2013). Numerous studies report elevated oxidative DNA damage in psychiatric populations, including increased levels of oxidized DNA nucleotides in peripheral body fluids, DNA strand breaks and mitochondrial DNA damage in peripheral blood leukocytes (Behnke et al., 2022; Czarny et al., 2019, 2015, 2018; Giménez-Palomo, Andreu, et al., 2024; Jorgensen et al., 2022; Morath et al., 2014; Palta, Samuel, Miller, & Szanton, 2014). For BPD, evidence remains limited but suggestive (Lee et al., 2020).

Notably, oxidative DNA damage and mitochondrial function are tightly interlinked. DNA damage triggers repair processes that can transiently decrease mitochondrial activity to mitigate further oxidative stress (Fang et al., 2016; Maynard et al., 2013). Mitochondria, in turn, modulate ROS production through regulation of proton leak and respiratory efficiency (Cheng et al., 2017; Demine et al., 2019; Fang et al., 2016; Zhao et al., 2019). Yet few studies have investigated the interplay between these cellular systems in psychiatric conditions. Research into chronic stress biology points to inverse associations between DNA integrity and mitochondrial capacity, activity, and density (Boeck et al., 2018; Czarny et al., 2019, 2018; Guillen-Parra et al., 2024; Mavioğlu et al., 2025), substantiating interest in their dynamic interactions in disorders such as BPD.

This case–control study is the first to directly examine mitochondrial bioenergetics and DNA damage in BPD. We assessed mitochondrial respiration and oxidative DNA damage in peripheral blood mononuclear cells (PBMCs) from women with acute BPD, remitted BPD, and healthy controls. We hypothesized that acute BPD would be characterized by reduced mitochondrial respiratory activity (e.g. less ATP production-related respiration) and elevated DNA damage compared to controls. Additionally, we explored whether these alterations differed between acute and remitted BPD and whether mitochondrial respiration and density were inversely associated with DNA damage across the entire cohort.

## Methods

### Study cohort

The study enrolled three groups matched for age and BMI: (a) 32 women with acute BPD meeting ≥5 DSM-5 criteria (American Psychiatric Association, 2013); (b) 15 women in remission from BPD, fulfilling ≤3 criteria, excluding self-harming behavior within two years prior to participation (Table 1); and (c) 29 women without a history of severe mental and somatic disorders. Participants were recruited as part of the DFG Clinical Research Unit 256 on BPD (Schmahl et al., 2014) at the Central Institute of Mental Health (CIMH) Mannheim and the Psychiatry Department of Heidelberg University, Germany.

**Table 1.** Sociodemographic and clinical characteristics of the study cohort

	*Med* (*IQR*)^a^			Group comparisons		
	Controls (*n* = 29)	Remitted BPD (*n* = 15)	Acute BPD (*n* = 32)	Test statistic^b^	*p*	Effect size
Age, years	25.5 (9.8)	32.0 (5.8)	26.0 (12.0)	χ^2^(2) = 3.52	.172	.022
Body mass index, kg/m^2^	22.8 (3.7)	21.5 (5.1)	21.9 (6.6)	χ^2^(2) = 0.42	.811	.000
Smoking, No. (%)	0 (0)	2 (13)	0 (0)	χ^2^(2) = 8.35	.038	.332
BPD symptoms (BSL–23)	1.0 (4.5)	12.5 (7.0)	45.0 (19.2)	χ^2^(2) = 55.78	<.001	.791
BPD criteria fulfilled (IPDE), No.	0.0 (0.0)	1.0 (2.8)	6.0 (2.0)	χ^2^(2) = 61.78	<.001	.842
Comorbid SCID diagnoses, No.	0.0 (0.0)	(0.0) 0.5	1.5 (1.0)	χ^2^(2) = 29.70	<.001	.379
Child maltreatment exposure (CTQ)	29.0 (3.5)	65.0 (15.0)	57.0 (22.8)	χ^2^(2) = 44.22	<.001	.650
Antidepressant medication, No. (%)	0 (0)	2^c^ (13)	7^d^ (22)	χ^2^(2) = 7.01	.016	.304

a Descriptive statistic given in *Med* (*IQR*), exception, count variables given in numbers and percentage.

b Data were analyzed with Kruskal–Wallis χ^2^ tests (effect size: η^2^_rank_) and Pearson χ^2^ tests for contingency tables (effect size: Cramer’s *V*) as appropriate.

c Medication, No.: sertraline (1), escitalopram (1).

d Medication, No.: venlafaxine (1), citalopram and sertraline (1), fluoxetine (2), escitalopram (3).

### Procedure

Patients and controls were recruited through online advertisements. Procedures adhered to the Declaration of Helsinki (World Medical Association, 2013) and were approved by the ethics committees of Heidelberg and Ulm Universities. After written informed consent, participants underwent standardized diagnostics by trained raters. BPD diagnoses were established using the International Personality Disorder Examination (IPDE) interview (Loranger et al., 1994). Comorbidities (Supplementary Table S1) were assessed with the German Structured Clinical Interview for DSM-IV Axis-I Disorders (SCID-I; Wittchen, Zaudig, & Fydrich, 1997) updated to DSM-5.

Exclusion criteria included: current pregnancy; lifetime diagnoses of schizophrenia spectrum, psychotic, and bipolar disorders; substance dependency in the past year; substance abuse in the past 2 months (excluding nicotine); epilepsy; and major somatic illness. All participants were free of psychotropic medication except for single individuals with BPD continuing their SSRIs/SNRIs regime during participation (Table 1). On-demand medications (e.g. tranquilizers, glucocorticoids) had to be paused for three days pre-assessment.

At blood sampling, self-report questionnaires served to assess current symptom severity, with BPD symptoms in the past week being quantified by the sum score of the 23-item Borderline Symptom List (BSL-23; Bohus et al., 2009) and the Zanarini Rating Scale (ZAN-BPS; Zanarini, 2003). Clinician-evaluated (IPDE) and self-reported BPD assessments showed high concordance (*r*_S_ = .83–.89, *p*’s < .001).

Risk factors and symptom domains were assessed in detail with a battery of German standardized questionnaires, including the Childhood Trauma Questionnaire (CTQ; Klinitzke, Romppel, Häuser, Brähler, & Glaesmer, 2012) to record maltreatment, abuse, and neglect experienced between 0 and 18 years of age; Beck’s Depression Inventory-II (BDI-II; Hautzinger, Keller, & Kühner, 2006); the State–Trait Anxiety Inventory (STAI; Laux, Glanzmann, Schaffner, & Spielberger, 1981); Symptom Checklist-90-Revised (SCL-90-R; Franke, 2002); the questionnaire of self-harming behavior (FSVV; Reicherzer & Brandl, 2011); the State–Trait Anger Expression Inventory-2 (STAXI-2; Rohrmann et al., 2013); Buss and Perry’s Aggression Questionnaire (AQ; Werner & Von Collani, 2004) assessing the inclination to frustration, physical and verbal aggression, and interpersonal hostility/mistrust; the Barratt Impulsiveness Scale (BIS-11; Preuss et al., 2008) measuring cognitive and motor impulsivity and lack of planning; and the Difficulties in Emotion Regulation Scale (DERS; Ehring, Svaldi, Tuschen-Caffier, & Berking, 2013) recording difficulties in recognizing and regulating negative emotions.

### Blood sampling and PBMC isolation

Approximately, 30 ml of peripheral venous whole blood (nonfasting) was collected into EDTA-buffered tubes (Sarstedt, Nümbrecht, Germany) between 10:00 a.m. and 3:00 p.m. under sterile conditions during medical rounds. Shortly thereafter, PBMCs were isolated using Ficoll-Paque density gradient centrifugation (GE Healthcare, Chalfont St. Giles, UK), washed three times in sterile phosphate-buffered saline (PBS; Invitrogen, USA) by centrifugation at 150 g for 10 minutes at room temperature (Heraeus Megafuge, Thermo Fisher Scientific, USA), and the resulting cell pellet was resuspended in ice-cold cryopreservation medium (dimethyl sulfoxide [DMSO] / fetal calf serum [FCS], Sigma-Aldrich, St. Louis, MO, USA; 1:10 dilution; <5 million PBMCs/ml) and stored at −80 °C for at least 6 hours in a prechilled isopropanol-filled cryocontainers (Nalgene, USA).

### Mitochondrial activity

All biological measurements were conducted by a single experimenter blinded to group assignment. PBMC aliquots were thawed, washed twice with pre-warmed PBS containing 2% FCS (Sigma Aldrich), and resuspended in 4.1 ml MiR-05 respiration medium (Oroboros Instruments). During preprocessing, 10 μl cell suspension was mixed with 10 μl trypan blue staining solution (Sigma-Aldrich) to count the total number of cells and to estimate the percentage of dead cells in the sample for oxygen consumption rate correction (pmol O_2_/sec × million living cells). Measurements were performed in duplicate at 37 °C using an Oxygraph-2 K (Oroboros Instruments, Austria). Immediately after loading and closing the oxygraphy chambers, 10 μl sodium pyruvate (2 M stock, Sigma-Aldrich) was added. A standard Substrate-Inhibitor-Uncoupler-Titration (SUIT) protocol was applied (Karabatsiakis et al., 2014), involving sequential addition of oligomycin, FCCP, rotenone, and antimycin A (Sigma-Aldrich) to record the mitochondrial functional states.

Routine respiration refers to the oxygen consumption of unstimulated cells and indicates basal OxPhos activity. Leak respiration, measured after complex V (ATP synthase) inhibition, reflects the residual respiration compensating for proton leak, slippage, and cation cycling across the mitochondrial membrane (Pesta & Gnaiger, 2012). Electron transfer (ET) capacity refers to maximal respiration rate not limited by complex V and after disruption of the mitochondrial proton gradient. Residual oxygen consumption, assessed after inhibition of all OxPhos-associated enzymes, was subtracted from all raw values to correct for non-OxPhos related cellular oxygen consumption and technical noise. Leak was subtracted from Routine to estimate ATP turnover-related respiration, and Routine subtracted from ET capacity defined the reserve capacity. Oxygen flux was recorded in real time using DatLab software 6.1.0.7 (Oroboros Instruments). Technical duplicates were averaged and normalized for living cell count (Pesta & Gnaiger, 2012). Coupling efficiency of ATP induction was derived as ATP turnover-related respiration relative to routine respiration.

### Mitochondrial content of cells

Intracellular mitochondrial network density of PBMCs was estimated via the activity of citrate synthase, a TCA pacemaker enzyme (Larsen et al., 2012). After respirometry, one million living cells were shock-frozen in liquid nitrogen and stored at −80 °C until analysis. Citrate synthase activity (CSA) was quantified spectrophotometrically (Ultrospec 2100 pro Photometer, Amersham Bioscience, Chicago, IL, USA) in duplicates as previously described (Boeck et al., 2016; Eigentler et al., 2012; Karabatsiakis et al., 2014).

### DNA damage

DNA damage was assessed via alkaline single-cell gel electrophoresis assay (comet assay) (Boeck et al., 2019; Singh, McCoy, Tice, & Schneider, 1988), quantifying single- and double-strand breaks and alkali-labile sites. About one million PBMCs per subject were thawed, washed in 9 ml PBS, and centrifuged at 1200 rpm for 10 min at 18 °C (Heraeus Megafuge, Thermo Fisher Scientific). After supernatant removal, the cell pellet was resuspended with 10 μl PBS and separated into duplicates (~75,000 cells). Each was mixed with 120 μl of 0.5% low melting-point agarose (Sigma-Aldrich) and applied to microscope slides (Thermo Fisher Scientific) pre-coated on one side with 1.5% medium electroendoosmosis agarose (Sigma-Aldrich). Coated slides were covered with a coverslip.

After solidification (4 min at 4 °C), the coverslip was removed and the slides were lysed overnight at 4 °C by immersion in a buffer (2.5 M NaCl, 100 mM EDTA, 10 mM Tris; Sigma-Aldrich) (p*H*: 10) with freshly added 1% Triton X-100 (Sigma-Aldrich) and 10% DMSO (Sigma-Aldrich). The slides were then immersed in the electrophoresis tank containing alkaline buffer (300 mM NaOH: VWR, Germany; 1 mM Na_2_H_2_EDTA: AppliChem PanReac, Germany; p*H* > 13) for 40 min before electrophoresis was performed at a constant voltage of 25 V (approx. 0.7 V/cm and 300 mA) at 4 °C for 40 min. Slides were neutralized, washed three times in 0.4 M Tris-base (p*H* 7.5), rinsed with distilled water, dehydrated in 99.8% ethanol for 5 min, and stored at room temperature until microscopy.

For quality control, each electrophoresis assay included X-ray irradiated (8 Gy) and untreated HeLa cells as a positive and negative control (data not shown). DNA on slides was stained with ethidium bromide (50 μl, 10 mg/ml; Carl Roth, Germany) and analyzed at 40-fold magnification using fluorescence microscopy (Olympus Lifescience BX41 microscope (Waltham, MA, USA) with a Basler scA1300 -32 fm camera (Soda Vision, Singapore) and a mercury vapor bulb with a 590 nm barrier filter and a 515–560 nm excitation filter (Zeiss, Germany)). Per subject, 200 cells (100 per duplicate slide) were randomly analyzed using Comet Assay IV software (Instem, UK). Median tail intensity (% DNA in tail, defined as the percentage ratio of fluorescence signal in the tail normalized to the fluorescence signal in the head of the comet assay) was used to quantify DNA damage, as it offers superior inter-batch and inter-laboratory reliability (Kumaravel, Vilhar, Faux, & Jha, 2009).

### Flow cytometry

Due to limited cell material, PBMC composition was analyzed after respirometry. The cell suspension gathered from oxygraph chambers was stained with propidium iodide (Miltenyi Biotec, Germany) to exclude dead cells using fluorescence-activated cell sorting (FACS) on a FACSAria III sorter (BD Biosciences, Germany). Antibodies (CD3 PE-Vio 770, CD4 APC, CD8 FITC, Miltenyi Biotec) identified helper (CD3^+^CD4^+^) and cytotoxic T cells (CD3^+^CD8^+^), and CD45RA^+^ antibodies (CD45RA PE, Miltenyi Biotec) distinguished naïve cells from memory cells. Quality control on randomly selected samples verified the homogeneity of the isolated subsets. Raw data were processed using BD FACSDiva 8.0.1 software (BD Biosciences). Cell counts (CD3^+^, CD3^+^CD4^+^, CD3^+^CD8^+^) were determined by fluorescence intensity levels in two-parameter fluorescence scattergrams, and percentages were calculated relative to viable CD3^+^ cells (Supplementary Table S2).

### Statistical analysis

Statistical analyses were performed using R 4.1.3 (R Core Team, 2024). Bivariate associations were examined using Spearman correlations (*r*_S_). Groups were compared using one-way Welch ANOVAs and Kruskal–Wallis tests, as appropriate, followed by *post hoc* tests (Games-Howell, Conover) reporting Cohen’s *d* and rank-biserial correlations *r*_X_ as effect size measures. Family-wise error rates were adjusted using Tukey or Holm corrections. All analyses used α < .050, two-tailed, as the threshold of statistical significance. Exploratory correlation analyses relied on 95% confidence intervals and are reported without *p*-values.

## Results

### Reduced mitochondrial energy production processes in BPD

**Figure 1.**
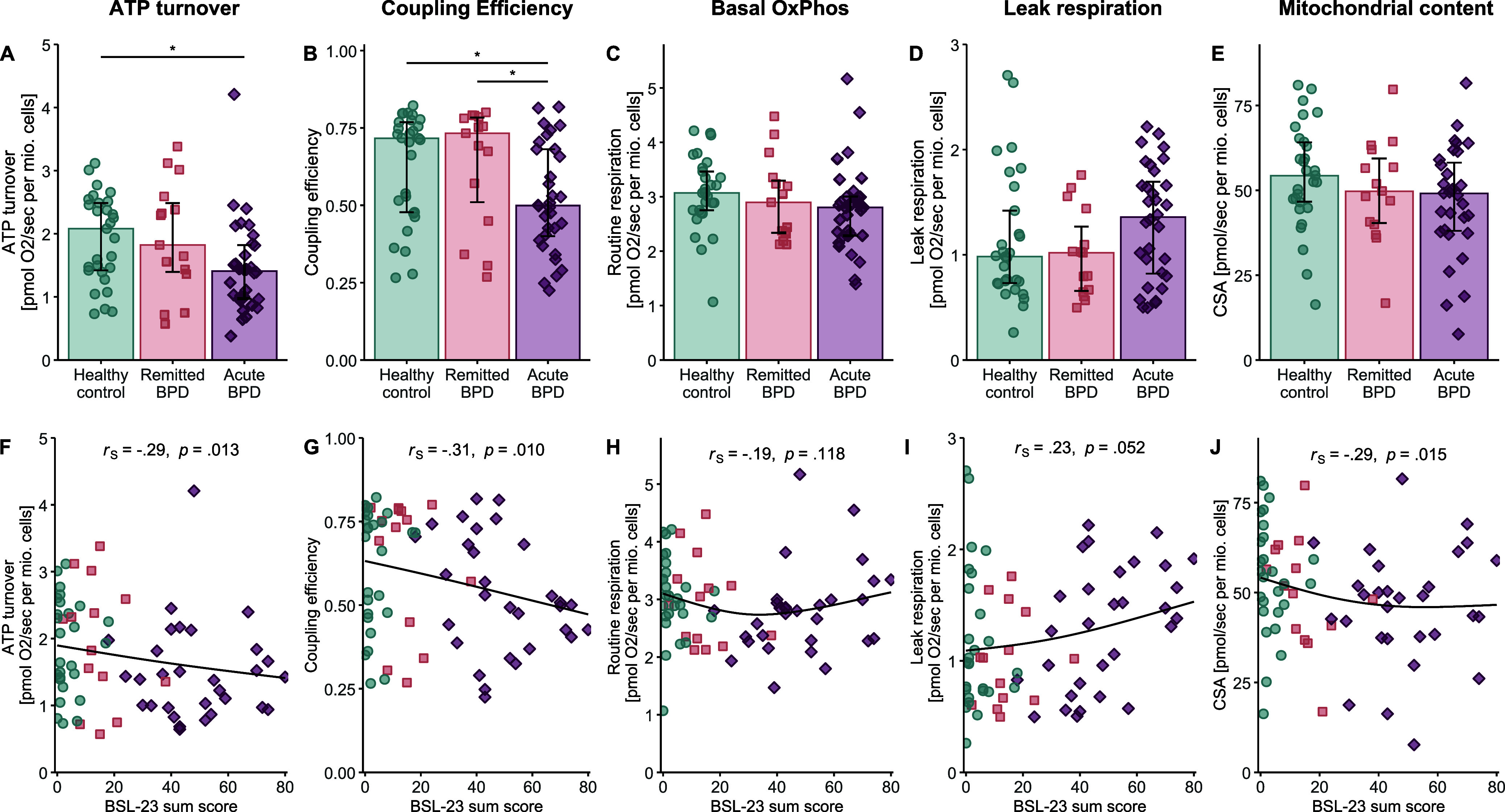
Group differences and associations of mitochondrial function and content in peripheral blood mononuclear cells (PBMCs). (a–e) Group comparisons of mitochondrial respiration parameters and mitochondrial content (CSA) across female healthy controls (*n* = 29, teal circles), and women with remitted BPD (*n* = 15, coral squares), and acute BPD (*n* = 32, bordeaux diamonds), matched for age and body mass index. Bar plots show median values with interquartile ranges. Group effects were assessed using Welch’s ANOVAs or Kruskal–Wallis tests, followed by *post hoc* pairwise comparisons, with significant comparisons being displayed, * *p_adj_* < .050. (f–j) Associations (Spearman’s *r*_S_) between self-reported BPD symptom severity, quantified with the Borderline Symptom List (BSL-23) sum score, and mitochondrial respiration parameters and CSA. Each point reflects an individual participant. We used natural cubic splines to illustrate the monotonic (but not necessarily linear) trends captured by Spearman’s correlation. CSA, citrate synthase activity; PBMCs, peripheral blood mononuclear cells; BPD, borderline personality disorder. Group colors refer to the online version (teal, coral, bordeaux), while a grayscale version (light gray, medium gray, dark gray) is shown in the print version.

Significant group differences were observed in mitochondrial ATP turnover (*F*(2,35.5) = 3.60, *p* = .038, η^2^ = .167) and coupling efficiency (i.e. ATP turnover relative to routine respiration, χ^2^(2) = 6.89, *p* = .032, η^2^_rank_ = .067) (Table 2). *Post hoc* analyses revealed that individuals with acute BPD had lower ATP turnover (*p*_adj_ = .037, Cohen’s *d* = −0.62, Figure 1a) and less efficient ATP production (coupling efficiency: *p*_adj_ = .029, *r*_x_ = −0.35, Figure 1b) compared to controls, indicating decreased mitochondrial energy production processes in acute BPD. Additionally, coupling efficiency was lower in acute BPD compared to remitted BPD (*p*_adj_ = .037, *r*_x_ = −0.36, Figure 1b). No significant group differences were found for basal OxPhos activity (Figure 1c, but see sensitivity analyses), leak respiration (Figure 1d), ET capacity, reserve capacity, and mitochondrial content in cells (Figure 1e) (Table 2).

**Table 2.** Group comparisons and correlations of mitochondrial parameters and DNA damage in peripheral blood mononuclear cells

	Controls	Remitted BPD	Acute BPD	Group comparisons			Spearman correlation, *r*_S_ (*p*)	
Variable	*M* (*SD*), *n* = 29	*M* (*SD*), *n* = 15	*M* (*SD*), *n* = 32	Test statistic^a^	*p*	η^2^	BPD symptoms^b^	DNA damage^c^
Routine respiration^d^	3.07 (0.68)	2.94 (0.76)	2.77 (0.79)	*F*(2,37.4) = 1.23	.304	.062	−.19 (.118)	.02 (.901)
Leak respiration^d^	1.14 (0.62)	1.01 (0.41)	1.31 (0.54)	χ^2^(2) = 3.23	.199	.017	.23 (.052)	.60^***^ (<.001)
Electron transfer capacity^d^	7.22 (2.50)	7.08 (2.01)	7.01 (2.67)	*F*(2,41.4) = 0.05	.950	.002	−.02 (.875)	.20 (.190)
ATP turnover-related respiration^d^^,^^e^	1.92 (0.70)	1.93 (0.89)	1.46 (0.73)	*F*(2,35.5) = 3.60	.038*	.167	−.29^*^ (.013)	−.41^**^ (.005)
Reserve capacity^d^	4.16 (2.04)	4.14 (1.42)	4.24 (2.11)	*F*(2,43.4) = 0.02	.979	.001	.03 (.793)	.21 (.162)
Coupling efficiency^f^	0.63 (0.18)	0.63 (0.19)	0.52 (0.17)	χ^2^(2) = 6.89	.032*	.067	−.31^**^ (.010)	−.57^***^ (<.001)
Citrate synthase activity^g^	54.30 (15.47)	49.97 (15.05)	46.94 (16.16)	*F*(2,38.4) = 1.61	.213	.077	−.29^*^ (.015)	−.28 (.066)
DNA damage^c^	3.26^h^ (3.33)	5.35^i^ (4.04)	4.71 (4.38)	χ^2^(2) = 3.04	.219	.016	.24 (.054)	−

*Note*: * *p* < .050, ^**^ *p* < .010, ^***^ *p* < .001, two-tailed.

**Figure 2.**
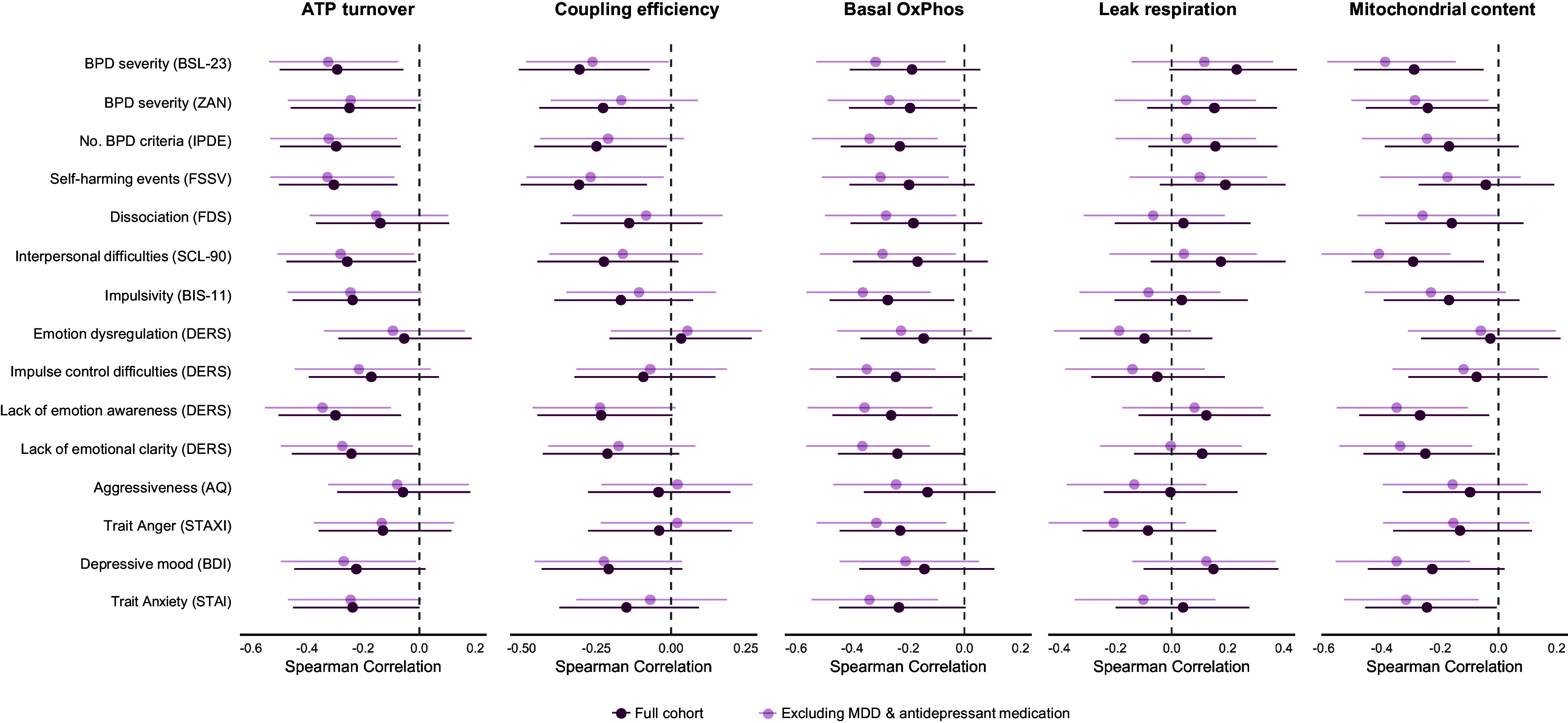
Exploratory correlations between mitochondrial parameters and borderline personality disorder (BPD) symptom domains. Forest plots display effect sizes (Spearman’s *r*_S_) and 95% confidence intervals for correlations between mitochondrial parameters assessed in peripheral blood mononuclear cells and clinical symptom domains related to BPD. Analyses were conducted in the full study sample (*N* = 76, bordeaux) and a sensitivity subsample excluding participants with current major depressive episodes and/or antidepressant medication use (*N* = 63, rosé). Symptom domains were assessed using standardized clinical questionnaires (see Methods). Due to the exploratory nature of the analyses, *p*-values were not computed. Each dot represents the correlation coefficient for a given symptom–mitochondrial parameter pair, and the bars indicate the 95% confidence interval. BPD, borderline personality disorder. Colors refer to the online version (bordeaux, rosé), while a grayscale version (dark gray, light gray) is shown in the print version.

a Data were analyzed with one-way Welch ANOVAs (*F*) or Kruskal–Wallis tests (χ^2^, effect size: η^2^_rank_) as appropriate.

b Severity of BPD symptoms was evaluated using the self-report questionnaires 23-item Borderline Symptom List (BSL-23). See Figure 2 for additional symptom measures.

c Indicated as median tail intensity (%DNA in tail) in the comet assay.

d in pmol O_2_/sec per million living cells.

e Games–Howell tests and effect sizes (Cohen’s *d*) for multiple group comparisons of ATP turnover-related respiration: acute BPD versus controls, *p*_adj_ = .037, *d* = −0.62; acute versus remitted BPD, *p*_adj_ = .200, *d* = −0.62; remitted BPD versus controls, *p*_adj_ > .999, *d* = 0.01.

f Conover tests and effect sizes (rank-biserial correlation *r*_x_) for multiple group comparisons of coupling efficiency: acute BPD versus controls, *p*_adj_ = .029, *r*_x_ = −0.35; acute versus remitted BPD, *p*_adj_ = .037, *r*_x_ = −0.36; remitted BPD versus controls: *p*_adj_ = .437, *r*_x_ = 0.04.

g in pmol/s per million living cells. One missing value.

h Values of three cases were missing due to insufficient cell material.

i Values of four cases were missing due to insufficient cell material.

To rule out that the observed effect pattern was influenced by comorbid episodes of MDD and/or antidepressant medication, we performed sensitivity analyses excluding such cases (Supplementary Table S3). These analyses confirmed our findings and additionally revealed that individuals with acute BPD exhibited decreased basal OxPhos activity (routine respiration: *p*_adj_ = .015, *d* = −0.80) and lower mitochondrial content in cells (CSA: *p*_adj_ = .017, *r*_x_ = −0.43) compared to controls (Supplementary Figure S1). Notably, visual inspections of boxplots suggested that acute BPD cases receiving antidepressant medication showed improved mitochondrial respiration compared to those without medication. Although this observation cannot be meaningfully statistically tested due to the low number of cases, the trend aligns with previous studies indicating that antidepressant could influence mitochondrial respiration in complex ways (Cikánková, Fišar, & Hroudová, 2020; Emmerzaal et al., 2021; Fernström et al., 2021).

### Symptom severity and mitochondrial function

Correlation analyses (Table 2) revealed that, regardless of diagnostic condition, higher BPD symptom severity was associated with reduced ATP turnover (*r*_S_ = −.29, *p* = .013, Figure 1f) and lower coupling efficiency (*r*_S_ = −.31, *p* = .010, Figure 1g). Basal OxPhos activity showed no significant association with BPD symptom severity in the entire cohort (Figure 1h); however, sensitivity analyses excluding individuals with concurrent MDD diagnosis and/or antidepressant medication revealed a negative association between BPD symptom severity and basal OxPhos activity (i.e. routine respiration: *r*_S_ = −.39, *p* = .002). There was a marginal association between greater symptom severity and higher leak respiration (*r*_S_ = .23, *p* = .052, Figure 1i) as well as a significant negative association between symptom load and mitochondrial content (*r*_S_ = −.29, *p* = .015, Figure 1j) in PBMCs. Sensitivity analyses confirmed these findings by yielding similar or stronger associations (Supplementary Table S3).

Furthermore, we explored the relationship between mitochondrial parameters and BPD symptom domains such as self-harming behavior, dissociative experiences, interpersonal difficulties, impulsivity, emotion dysregulation, trait anger, and depressed mood, as assessed by standardized clinical questionnaires. As summarized in Figure 2, mitochondrial indices related to energy metabolism (basal OxPhos activity, ATP turnover, coupling efficiency) and cellular content showed mostly negative correlations with broad traits and specific symptom domains. The individual associations were generally small (|*r*_S_| ≤ 0.34) with various confidence intervals including zero; however, the consistency in direction and conceptual alignment across symptom domains suggests a coherent association pattern rather than random fluctuation. This pattern was also observed in the sensitivity subsample excluding cases with comorbid MDD episodes and/or antidepressant medication, further suggesting that the observed associations are not limited to depressive symptoms.

### DNA damage and mitochondrial function

**Figure 3.**
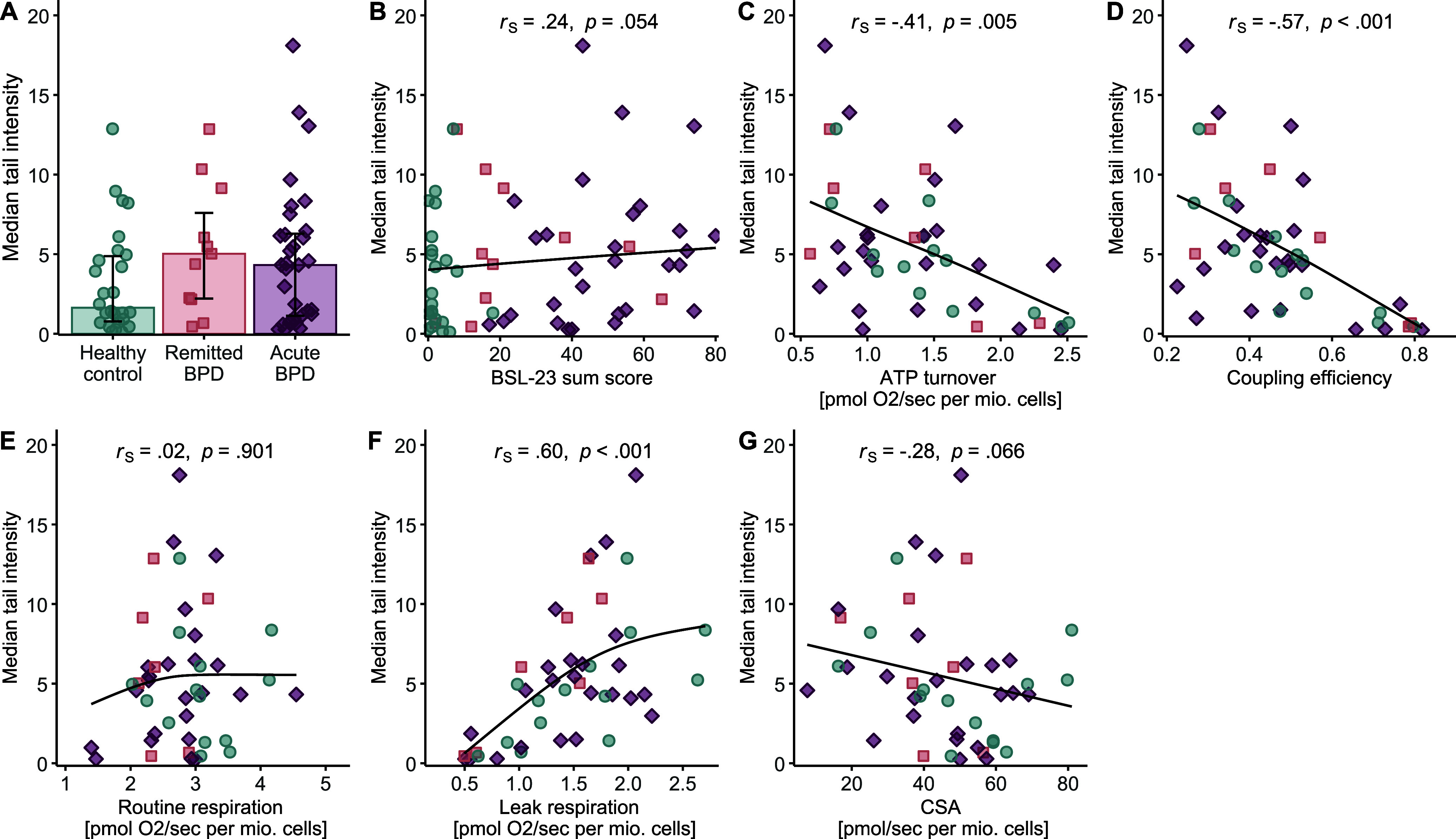
Group differences and associations of DNA damage in peripheral blood mononuclear cells (PBMCs). (a) Group comparison of DNA damage, quantified as medium tail intensity in the comet assay between female healthy controls (*n* = 26, teal circles), and women with remitted BPD (*n* = 11, coral squares), and acute BPD (*n* = 32, bordeaux diamonds), matched for age and body mass index. Bar plots show median values with interquartile ranges. Group effects were assessed using a Kruskal–Wallis test. (b) Bivariate association (Spearman’s *r*_S_) between self-reported BPD symptom severity, quantified with the Borderline Symptom List (BSL-23) sum score, and medium tail intensity in PBMCs. (c–g) Associations (Spearman’s *r*_S_) between DNA damage and mitochondrial respiration parameters (c–f), assessed with high-resolution respirometry, and mitochondrial content (g), quantified as citrate synthase activity (CSA). Each point reflects an individual participant. We used natural cubic splines to illustrate the monotonic (but not necessarily linear) trends captured by Spearman’s correlation. CSA, citrate synthase activity; PBMCs, peripheral blood mononuclear cells; BPD, borderline personality disorder. Group colors refer to the online version (teal, coral, bordeaux), while a grayscale version (light gray, medium gray, dark gray) is shown in the print version.

DNA damage levels did not significantly differ between groups (Kruskal–Wallis χ^2^(2) = 3.04, *p* = .219, η^2^_rank_ = .016; Table 2; Figure 3a). There was a trend suggesting that higher BPD symptom severity was associated with greater DNA damage (*r*_S_ = .24, *p* = .054; Figure 3b). We also tested whether DNA damage was inversely related to mitochondrial bioenergetics. Indeed, DNA damage was negatively correlated with ATP turnover (*r*_S_ = −.41, *p* = .005, Figure 3c) and coupling efficiency (*r*_S_ = −.57, *p* < .001, Figure 3d), but not basal OxPhos activity (Figure 3e). Additionally, DNA damage was positively correlated with leak respiration (*r*_S_ = .60, *p* < .001, Figure 3f), and there was a negative association between DNA damage and mitochondrial content in trend (*r*_S_ = −.28, *p* = .066, Figure 3g). The pattern of findings remained unchanged when excluding cases with a comorbid MDD episodes and/or antidepressant medication (Supplementary Table S3).

Age and BMI were not associated with mitochondrial markers and DNA damage. Childhood maltreatment exposure (CTQ scores) was not significantly associated with mitochondrial or DNA markers (all |*r*_S_| < .14, all *p* > .247), although individuals with lifetime BPD reported significantly higher CTQ scores than controls (see Table 1). Statistical adjustment of the main analyses via ANCOVA was not appropriate, as the strong confounding between BPD diagnosis and CTQ scores (e.g. ≥60% of BPD patients versus 7% of controls with moderate-to-severe exposure) violates the assumption of covariate independence (Miller & Chapman, 2001).

## Discussion

Individuals with acute BPD showed reduced mitochondrial energy production activity in PBMCs compared to controls, specifically characterized by lower oxygen consumption devoted to ATP production and reduced ATP production efficiency. When excluding participants with comorbid MDD episodes and/or antidepressant use, we also observed reductions in basal OxPhos activity and mitochondrial content, altogether suggesting a downregulated density, activity, and ATP supply of mitochondria in acute BPD. These alterations were not associated with the lifetime diagnosis of BPD but appeared more closely related to current symptom severity. This aligns with findings in bipolar disorder and MDD, supporting the emerging view that peripheral mitochondrial alterations may reflect acute psychopathology and may be sensitive to phase transitions and remission (Allen, Romay-Tallon, Brymer, Caruncho, & Kalynchuk, 2018; Czarny et al., 2015, 2018; Gamradt et al., 2021; Giménez-Palomo, Guitart-Mampel, et al., 2024; Gumpp et al., 2021; Karabatsiakis et al., 2014; Ni et al., 2024; Papageorgiou & Filiou, 2024; Triebelhorn et al., 2022).

Importantly, our sensitivity analyses suggested that the observed mitochondrial alterations in acute BPD were not attributable to comorbid depression or antidepressant medication. Furthermore, exploratory correlation analyses illustrated that reduced mitochondrial activity may be related to a broader range of core BPD symptom domains beyond depressive symptoms, such as self-harming behavior, interpersonal difficulties, impulsivity, and emotion regulation problems. Although the direction of associations was broadly coherent across BPD symptom domains, the individual effects were generally small in size (|*r*_S_| ≤ 0.34) and should be considered hypothesis-generating, warranting replication in larger cohorts.

ET capacity and reserve capacity were preserved in acute BPD, suggesting no functional impairment of the electron transport chain function. Rather than a mitochondrial “dysfunction”, the observed bioenergetic profile likely reflects a downregulation in mitochondrial activity and density. Such a regulatory response may serve multiple purposes, including to limit ROS production under stress conditions. Diminishing the ROS induction can be achieved via increasing in proton leak across the inner mitochondrial membrane, which also lowers the efficiency of ATP production (Cheng et al., 2017; Demine et al., 2019; Zhao et al., 2019). While we observed no group differences in mitochondrial leak, leak respiration correlated positively with BPD symptom severity, suggesting that this parameter may be transiently upregulated with acute symptom severity.

Maintaining a balance between ATP production and ROS induction is vital for cell survival. Oxidative stress can damage key cell components, including mitochondrial and nuclear DNA (Czarny et al., 2018; Fang et al., 2016; Kidane et al., 2014). Previous studies have reported heightened cellular and systemic oxidative stress and reduced antioxidative defense in BPD (Díaz-Marsá et al., 2012; Lee et al., 2020; MacDowell et al., 2020; Ruiz-Guerrero et al., 2023), consistent with elevated oxidative stress markers and DNA damage established in other psychiatric disorders (Behnke et al., 2022; Czarny et al., 2015, 2018; Morath et al., 2014). While we did not find group-level differences in DNA damage, marginal positive associations with symptom severity and robust correlations with mitochondrial measures indicate biological relevance. The lack of group effects may reflect a type II error, due to the smaller sample size for DNA assays caused by limited cell material.

Specifically, greater DNA damage was associated with lower mitochondrial efficiency and ATP production, as well as increased (proton) leak. These findings align with evidence that DNA damage activates repair mechanisms that transiently suppress mitochondrial biogenesis and coupling to minimize further oxidative stress during phases of DNA repair (Cheng et al., 2017; Demine et al., 2019; Zhao et al., 2019). DNA repair processes—particularly poly(ADPribose) polymerase 1 (PARP1) activity—can directly diminish mitochondrial activity through depletion of nicotinamide adenine nucleotide (NAD^+^) and indirectly modulate mitochondrial biogenesis and mitophagy via transcriptional pathways (Fang et al., 2016; Murata et al., 2019; Thomas et al., 2018). In line with this, we observed negative associations between DNA damage and mitochondrial ATP coupling, as well as a marginal negative association between DNA damage and mitochondrial content (*p* = .066). Notably, the observed reduction in OxPhos activity and ATP production—but not efficiency—could be accounted for by lower mitochondrial content. Potential upstream mechanisms such as oxidative damage-induced modulation of mitophagy and mitochondrial biogenesis warrant further investigation. Particularly relevant in this context may also be damage to and repair of mitochondrial DNA (mtDNA), with initial studies reporting elevated mtDNA damage and altered repair efficiency in chronic stress and psychiatric disorders such as schizophrenia, bipolar disorder, and MDD (Czarny et al., 2018, 2019, 2020).

The observed connection between DNA damage and mitochondrial activity and density also adds to studies indicating that mitochondrial alterations are linked to compromised maintenance of telomeric DNA in the context of chronic psychosocial stress. Recent studies in cohorts with chronic stress and early adversity highlighted associations between stress exposure, prematurely shortened telomeres, and stress-associated decrease in mitochondrial respiratory capacity, activity, efficiency, and density (Boeck et al., 2018; Guillen-Parra et al., 2024; Mavioğlu et al., 2025). These patterns warrant further efforts in understanding the relevance of the reciprocal regulation of DNA/telomere integrity and mitochondrial dynamics for health and aging under stress (Czarny et al., 2019, 2018; Mavioğlu et al., 2025).

Our results add to growing evidence for complex energy metabolism alterations as a convergent biological pathway across psychiatric disorders (Andreazza et al., 2025; Ni et al., 2024; Papageorgiou & Filiou, 2024). Chronic stress is likely a key driver of persistent metabolic shifts (Bobba-Alves et al., 2023; Boeck et al., 2016; Guillen-Parra et al., 2024; Gumpp et al., 2020; Mavioğlu et al., 2025), linking psychosocial adversity to the multifaceted molecular signatures observed in psychiatric disorders such as oxidative stress, impaired DNA/telomere maintenance, and chronic cytokine activity (Behnke et al., 2022; Bernard et al., 2025; Boeck et al., 2018; Czarny et al., 2019; Darrow et al., 2016; Jorgensen et al., 2022; Morath et al., 2014; Yuan, Chen, Xia, Dai, & Liu, 2019), which might even diminish individual treatment responses (Strawbridge et al., 2015). Future studies should investigate these mechanisms longitudinally at multiple layers of the biological system, including *in vitro* models exposing cells to energetic challenges such as stress hormones and DNA damage (Behnke et al., 2022; Bobba-Alves et al., 2023; Czarny et al., 2020; Thomas et al., 2018), and *in vivo* physical and psychosocial stress paradigms comparing patient and control groups (Kelly et al., 2024; Moreno-Villanueva et al., 2019). These approaches will advance our mechanistic understanding of mitochondrial regulation and connected biological processes under stress and their role in disease formation, progression, and treatment.

## Limitations

This study has limitations, including its female-only cohort, which may limit generalizability beyond women. A key limitation is the use of nonfasting, variably timed blood samples, likely introducing unsystematic variance and reducing sensitivity to detect group differences, though unlikely to have generated spurious effects. Future studies should implement fasting morning protocols.

Due to the high overlap between BPD and childhood maltreatment, statistical adjustment for early adversity was not feasible. Although prior work suggests links between maltreatment and mitochondrial function (e.g. Gumpp et al., 2023, 2020; Mavioğlu et al., 2025), no such associations emerged in our sample. Future studies should stratify for early adversity to separate diagnostic from developmental influences.

PBMC subtype composition may also affect mitochondrial measures, as subtypes differ in content and activity (Gamradt et al., 2021; Rausser et al., 2021). While PBMC composition did not systematically differ across groups, we observed a trend toward increased CD4^+^ memory T cells in acute BPD compared to controls (*p*_adj_ = .059), and these cells were associated with higher DNA damage and lower mitochondrial coupling (Supplementary Tables S2 and S4). Future research should investigate metabolic profiles of isolated immune cell types.

While our sensitivity analyses accounted for antidepressant use, medication effects on mitochondrial function are complex and heterogenous across drug classes (Cikánková et al., 2020; Emmerzaal et al., 2021; Fernström et al., 2021), with potential implications for mitochondrial profiles in (poly-)medicated cohorts. Lastly, the cross-sectional design limits causal interpretation of the associations between biological and clinical measures.

## Conclusions

This study provides the first evidence of reduced mitochondrial energy metabolism in peripheral immune cells from individuals with acute BPD. These alterations appear transient and disease state-dependent, normalizing with symptom remission. Our finding adds to growing evidence suggesting mitochondrial bioenergetics as a sensitive peripheral marker of disorder severity. Further work is needed to elucidate the causes and consequences of mitochondrial downregulation and its interplay with DNA damage in the context of chronic stress and psychopathology. Unraveling metabolic disruptions in brain and periphery will inform innovative disease models and treatment approaches for severe psychiatric disorders.

## Supporting information

Behnke et al. supplementary material Behnke et al. supplementary material

## Supplementary material

The supplementary material for this article can be found at http://doi.org/10.1017/S0033291725102493.
