# Supplementary material for: Mitochondrial respiratory activity and DNA damage in peripheral blood mononuclear cells in borderline personality disorder
Source: Psychol Med. 2025 Nov 28;55:e365. doi: 10.1017/S0033291725102493 (PMC13058628; doi:10.1017/S0033291725102493)
Supplement: Behnke et al. supplementary material [file S0033291725102493sup001.pdf]

Supplementary Material

Mitochondrial respiratory activity and DNA damage in peripheral blood mononuclear cells in borderline personality disorder

Authors: Alexander Behnke*, Manuela Rappel, Laura Ramo-Fernández, R. Nehir Mavioğlu, Benjamin Weber, Felix Neuner, Ellen Bisle, Matthias Mack, Peter Radermacher, Stephanie H. Witt, Christian Schmahl, Alexander Karabatsiakis, Iris-Tatjana Kolassa*

* *Corresponding authors*: Dr. Alexander Behnke, e-mail: alexander.behnke@uni-ulm.de; Prof. Dr. Iris-Tatjana Kolassa, e-mail: iris.kolassa@uni-ulm.de, Phone: +49 731 5026 591, Fax: +49 731 5026 599

Supplementary Tables

Supplementary Table 1. Characterization of the cohort's psychiatric comorbidity.

	No. (%)			Test statistic		
	Controls (*n*=29)	Remitted BPD (*n*=15)	Acute BPD (*n*=32)	$\chi^2_{Pearson}$(df)	p_{Fisher}	Cramer's *V*
Major depressive disorder (F32.x/F33.x)	0 (0)	0 (0)	6[a] (19)	9.37 (2)	.014	.358
Dysthymic disorder (F34.1)	0 (0)	1 (7)	7[a] (22)	7.19 (2)	.015	.333
Adjustment disorder	0 (0)	0 (0)	1 (3)	1.39 (2)	.999	.135
Posttraumatic stress disorder	0 (0)	0 (0)	10 (45)	15.83 (2)	<.001	.456
Panic disorder	0 (0)	0 (0)	8 (25)	12.29 (2)	.002	.402
Agora phobia	1 (3)	1 (7)	1 (3)	0.37 (2)	.790	.070
Social phobia	1 (3)	0 (0)	10 (31)	12.66 (2)	.002	.408
Specific phobia	1 (3)	0 (0)	2 (6)	1.08 (2)	.999	.119
Obsessive-compulsive disorder	3 (10)	1 (7)	1 (3)	1.29 (2)	.508	.130
Bulimia nervosa	0 (0)	1 (7)	5 (16)	5.15 (2)	.077	.260
Binge eating disorder	0 (0)	0 (0)	2 (6)	2.82 (2)	.676	.193
Somatization disorder	1 (3)	0 (0)	0 (0)	1.64 (2)	.598	.147
Nicotine consumption	0 (0)	2 (13)	0 (0)	8.35 (2)	.038	.332

Note: [a] including two cases with double depression (F33.x and F34.1). Diagnoses not listed in the table were not present among participants.

Supplementary Table 2. Blood cell viability and composition of thawed immune cell samples.

	***M* (*SD*)**			**Group comparisons**		
	Controls (n = 29)	Remitted BPD (n = 15)	Acute BPD (n = 32)	Welch ANOVA $F(df)$	p	η^2
Viability of thawed cells in total PBMCs, %	83.0 (7.4)	85.7 (4.0)	84.9 (6.3)	1.14 (2, 42.69)	.329	.051
$CD3^+$ T cells, %[a]	48.0 (12.3)	47.0 (14.6)	53.8 (15.8)	1.47 (2, 36.45)	.242	.075
$CD4^+$ naïve T cells, %[b]	17.1 (10.6)	20.8 (15.2)	18.8 (10.8)	0.39 (2, 34.00)	.677	.023
$CD4^+$ memory T cells, %[b,c]	15.9 (9.3)	15.9 (10.2)	21.1 (7.3)	3.35 (2, 34.49)	.047	.163
$CD8^+$ naïve T cells, %[b]	16.0 (6.3)	14.8 (8.7)	12.8 (4.8)	2.30 (2, 32.52)	.116	.124
$CD8^+$ memory T cells, %[b]	5.1 (4.1)	5.1 (4.4)	7.6 (5.1)	2.50 (2, 37.69)	.096	.117

Note: [a] Percentage of cell types of total living PBMCs after respirometry.

[b] Percentage of total viable T cells ($CD3^+$) after respirometry.

[c] Games-Howell tests and effect sizes (Cohen's *d*) for multiple group comparisons of the percentage of $CD4^+$ memory T cells of total viable T cells ($CD3^+$) after respirometry: acute BPD vs. controls, p_{adj} = .059, d = -0.59; acute vs. remitted BPD, p_{adj} = .206, d = -0.60; remitted BPD vs. controls, p_{adj} > .999, d < 0.01.

Supplementary Table 3. Sensitivity analysis of mitochondrial parameters and DNA damage in peripheral blood mononuclear cells under exclusion of cases with current major depression episode and/or antidepressant medication.

	**Controls**	**Remitted BPD**	**Acute BPD**	**Group comparisons**			**Spearman correlation, r_S (*p*)**	
**Variable**	*M* (*SD*), *n* = 29	*M* (*SD*), *n* = 13	*M* (*SD*), *n* = 21	Test statistic[a]	*p*	η^2	BPD symptoms[b]	DNA damage[c]
Routine respiration[d,e]	3.07 (0.68)	2.88 (0.65)	2.57 (0.54)	*F*(2, 31.4) = 0.24	.024*	.211	-.39** (.002)	-.01 (.944)
Leak respiration[d]	1.14 (0.62)	0.96 (0.42)	1.27 (0.55)	$\chi^2(2) = 2.29$	.319	.005	.14 (.298)	.62*** (<.001)
Electron transfer capacity[d]	7.22 (2.50)	6.95 (2.02)	6.34 (2.13)	*F*(2, 33.4) = 0.93	.406	.053	-.20 (.135)	.15 (.406)
ATP turnover-related respiration[d,f]	1.92 (0.70)	1.92 (0.77)	1.30 (0.47)	*F*(2, 29.5) = 8.39	.001**	.363	-.41** (.001)	-.50** (.002)
Reserve capacity[d]	4.16 (2.04)	4.07 (1.50)	3.77 (1.75)	*F*(2, 34.4) = 0.27	.764	.015	-.11 (.413)	.15 (.396)
Coupling efficiency[g]	0.63 (0.18)	0.65 (0.18)	0.52 (0.17)	$\chi^2(2) = 8.05$	.018*	.101	-.35** (.007)	-.65*** (<.001)
Citrate synthase activity[h,i]	54.30 (15.47)	48.69 (13.20)	42.90 (14.88)	$\chi^2(2) = 6.38$	.041*	.074	-.49*** (<.001)	-.32 (.062)
DNA damage[c]	3.26[j] (3.33)	5.38[j] (4.25)	4.70 (5.06)	$\chi^2(2) = 2.28$	.320	.005	.20 (.161)	

Note: * $p < .050$, ** $p < .010$, *** $p < .001$, two-tailed.

[a] Data were analyzed with one-way Welch ANOVAs (*F*) or Kruskal-Wallis tests (χ^2) as appropriate.

[b] Severity of BPD symptoms was evaluated using the self-report questionnaires 23-item Borderline Symptom List (BSL-23). See Figure 2 for additional symptom measures.

[c] Indicated as median tail intensity (%DNA in tail) in the comet assay.

[d] in pmol O_2/sec per million living cells.

[e] Games-Howell tests and effect sizes (Cohen's *d*) for multiple group comparisons of routine respiration (i.e. basal OxPhos activity): acute BPD vs. controls, $p_{adj} = .015$, $d = -0.80$; acute vs. remitted BPD, $p_{adj} = .322$, $d = -0.51$; remitted BPD vs. controls, $p_{adj} = .684$, $d = -0.29$.

[f] Games-Howell tests and effect sizes (Cohen's *d*) for multiple group comparisons of ATP-turnover related respiration: acute BPD vs. controls, $p_{adj} < .001$, $d = -0.96$; acute vs. remitted BPD, $p_{adj} = .044$, $d = -0.96$; remitted BPD vs. controls, $p_{adj} > .999$, $d = -0.01$.
[g] Conover tests and effect sizes (rank-biserial correlation r_x) for multiple group comparisons of coupling efficiency: acute BPD vs. controls, $p_{adj} = .019$, $r_x = -0.41$; acute vs. remitted BPD, $p_{adj} = .013$, $r_x = -0.49$; remitted BPD vs. controls, $p_{adj} = .313$, $r_x = 0.09$.
[h] in pmol/s per million living cells. One missing value.
[i] Conover tests and effect sizes (rank-biserial correlation r_x) for multiple group comparisons of citrate synthase activity: acute BPD vs. controls, $p_{adj} = .017$, $r_x = -0.43$; acute vs. remitted BPD, $p_{adj} = .249$, $r_x = -0.23$; remitted BPD vs. controls, $p_{adj} = .151$, $r_x = -0.19$.
[j] Values of 3 cases were missing due to insufficient cell material.

Supplementary Table 4. Bivariate associations of mitochondrial parameters and DNA damage assessed in bulk peripheral blood mononuclear cells (PBMCs) with the proportion of PBMC subpopulations.

	% CD3 T cells	% CD4 naïve T cells	% CD4 memory T cells	% CD8 naïve T cells	% CD8 memory T cells
Routine respiration	-.24* (.040)	-.12 (.306)	-.12 (.333)	-.25* (.037)	-.20 (.084)
Leak respiration	.39*** (<.001)	.26* (.028)	.64*** (<.001)	-.14 (.245)	.61*** (<.001)
Electron transfer capacity	-.10 (.405)	.12 (.302)	-.07 (.561)	-.01 (.943)	-.08 (.487)
ATP turnover-related respiration	-.50*** (<.001)	-.29* (.013)	-.55*** (<.001)	-.11 (.371)	-.59*** (<.001)
Reserve capacity	-.02 (.883)	.21 (.073)	-.05 (.673)	.09 (.442)	-.03 (.776)
Coupling efficiency	-.50*** (<.001)	-.30* (.010)	-.65*** (<.001)	-.01 (.970)	-.65*** (<.001)
Citrate synthase activity	-.39*** (<.001)	-.29* (.014)	-.37** (.001)	-.21 (.078)	-.34** (.003)
DNA damage	.15 (.292)	.13 (.365)	.34* (.017)	-.15 (.314)	.31* (.026)

Note: * $p < .050$, ** $p < .010$, *** $p < .001$, two-tailed significance of Spearman correlations (*p*-values in brackets). Cell counts represent percentage of cells types of total living PBMCs after respirometry.

Supplementary Figures

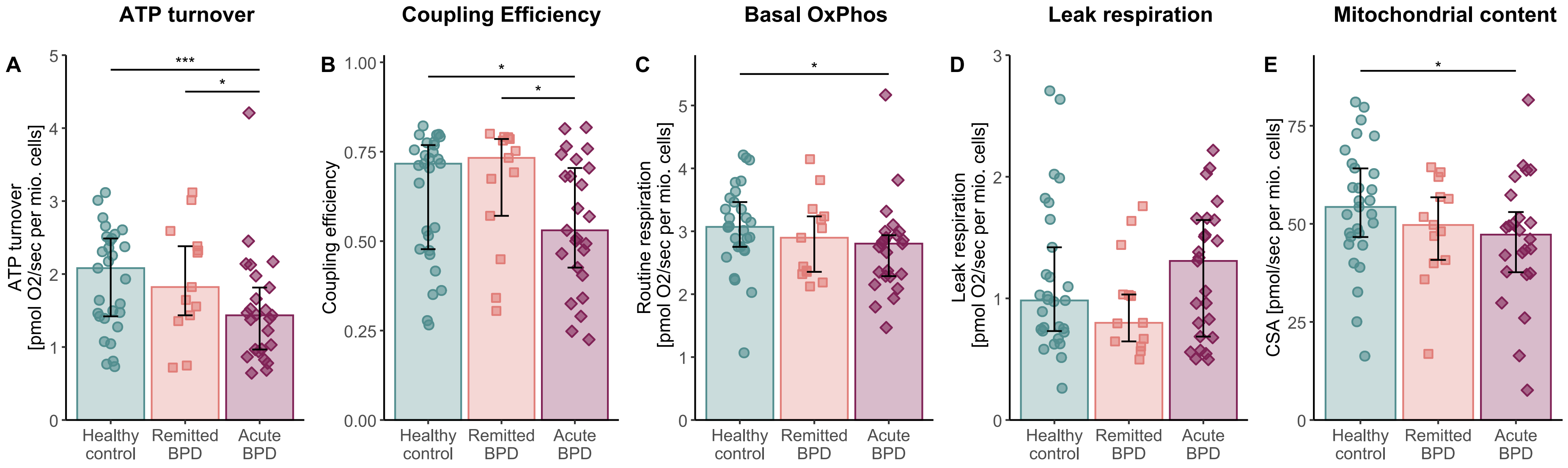

Supplementary Figure 1. Group differences in mitochondrial function and content in peripheral blood mononuclear cells (PBMCs), excluding participants with current major depressive episodes and/or antidepressant medication. (A–D) Group comparisons of mitochondrial respiration parameters and mitochondrial content (citrate synthase activity [CSA]) across female healthy controls (*n* = 29, teal circles) and women with remitted BPD (*n* = 13, coral squares) and acute BPD (*n* = 21, bordeaux diamonds). Bar plots display median values with interquartile ranges. Group differences were assessed using Welch's ANOVAs or Kruskal-Wallis tests, followed by *post hoc* pairwise comparisons. Significant comparisons are indicated as follows: $^{*}\ p_{adj} < .050$, $^{***}\ p_{adj} < .001$. CSA, citrate synthase activity; PBMCs, peripheral blood mononuclear cells; BPD, borderline personality disorder.
